# Quantum Chemical Approaches in Structure-Based Virtual Screening and Lead Optimization

**DOI:** 10.3389/fchem.2018.00188

**Published:** 2018-05-29

**Authors:** Claudio N. Cavasotto, Natalia S. Adler, Maria G. Aucar

**Affiliations:** Laboratory of Computational Chemistry and Drug Design, Instituto de Investigación en Biomedicina de Buenos Aires, CONICET, Partner Institute of the Max Planck Society, Buenos Aires, Argentina

**Keywords:** quantum mechanics, semi-empirical methods, structure-based drug design, molecular docking, drug lead optimization, binding free energy, molecular dynamics

## Abstract

Today computational chemistry is a consolidated tool in drug lead discovery endeavors. Due to methodological developments and to the enormous advance in computer hardware, methods based on quantum mechanics (QM) have gained great attention in the last 10 years, and calculations on biomacromolecules are becoming increasingly explored, aiming to provide better accuracy in the description of protein-ligand interactions and the prediction of binding affinities. In principle, the QM formulation includes all contributions to the energy, accounting for terms usually missing in molecular mechanics force-fields, such as electronic polarization effects, metal coordination, and covalent binding; moreover, QM methods are systematically improvable, and provide a greater degree of transferability. In this mini-review we present recent applications of explicit QM-based methods in small-molecule docking and scoring, and in the calculation of binding free-energy in protein-ligand systems. Although the routine use of QM-based approaches in an industrial drug lead discovery setting remains a formidable challenging task, it is likely they will increasingly become active players within the drug discovery pipeline.

## Introduction

The drug discovery process relied for many years on the experimental high-throughput screening of large chemical libraries to identify and optimize new drug lead compounds. In spite of efforts to improve its efficiency, this remained an expensive and time consuming process (Phatak et al., 2009). The availability of 3D structures of protein-ligand (PL) complexes has guided lead optimization for many years, paving the way to a more rational approach. Later on, theoretical developments, coupled with better computational algorithms and faster computing resources, allowed the routine use of *in silico* methods to model PL interaction, estimate binding affinity, and screen chemical libraries using structure-based approaches. Today, computational chemistry is a well-established and valuable tool in the drug discovery process (Cavasotto and Orry, 2007; Jorgensen, 2009).

The central quantity in PL association is the binding free energy (Δ*G*_*binding*_), a property of enormous relevance in the pharmaceutical industry, and no effort is too great to accurately estimate it in a computationally efficient way. Reliable prediction of receptor-small-molecule affinities in the early-stages of the drug discovery pipeline would be instrumental to rationally design new, more potent, and safer drugs, saving precious effort, time and cost. The accurate calculation of Δ*G* depends on several factors: (i) the energy model of the system; (ii) the accounting for protein flexibility; (iii) the presence of water molecules within the binding site and the solvation model. The last two challenges have been thoroughly addressed in recent reviews [(Cavasotto, 2011, 2012b; Spyrakis et al., 2011), and (Spyrakis and Cavasotto, 2015), respectively]. The last 20 years have seen a remarkable advance in theoretical and algorithmic developments for the calculation of binding affinities (Gohlke and Klebe, 2002; Gilson and Zhou, 2007; Mobley and Gilson, 2017), ranging from fast estimates, to be used in high-throughput docking and scoring (Cross et al., 2009; Cavasotto, 2012a), to much slower—yet more accurate—calculations using free energy perturbation or thermodynamic integration (Mobley and Klimovich, 2012; Hansen and Van Gunsteren, 2014), well-suited to guide chemical synthesis for hit-to-lead optimization. Most of these applications have been rooted in molecular mechanics (MM) force-fields (FF), but recent years have seen the development and application of quantum mechanical (QM) methods to biomacromolecular systems in the context of drug lead discovery and design. The recent blind challenges for ligand-pose and binding affinity predictions ran by the Drug Design Data Resource (D3R) in 2015 (Gathiaka et al., 2016) and 2016 (Gaieb et al., 2018) highlight the critical relevance of method development and benchmarking in pose prediction and affinity ranking of bound ligands.

It should be highlighted that the QM formulation accounts for all contributions to the energy (including effects missing in FFs, such as electronic polarization, charge transfer, halogen bonding, and covalent-bond formation), and thus is, in principle, theoretically exact; moreover, it offers the advantage of being general across the chemical space, avoiding system-dependent parameterizations, so that all elements and interactions can be considered on equal footing. In fact, QM has been present since the early days of computer-aided drug design (cf. the pioneering work of W.G. Richards on quantum pharmacology; Richards, 1977), and it is routinely used to derive FF parameters [such as torsional potentials from high level *ab initio* data and partial atomic charges by fitting to electrostatic surface potentials (ESP) (Mucs and Bryce, 2013)], in QSAR methods (De Benedetti and Fanelli, 2014), to study reaction mechanisms (Blomberg et al., 2014), and small-molecule strain (Forti et al., 2012; Juárez-Jiménez et al., 2015).

The goal of this short mini-review is to highlight the growing importance of quantum chemistry (QC) in the study of PL interaction, and present the latest applications of explicit QM calculations to structure-based drug design in the context of lead identification and optimization [for a survey on the development rather than application of QM methods for ligand-binding affinity calculations the reader is referred to an excellent recent review (Ryde and Söderhjelm, 2016); the review by Korth also offers a comprehensive coverage on the development of semiempirical QM and density functional theory (DFT) methods augmented by hydrogen-bonding and dispersion corrections (Yilmazer and Korth, 2016)].

## Quantum chemical approaches in protein-ligand docking

*In silico* molecular docking has been widely used to determine the binding mode (pose) of small-molecules to a binding site. However, the true potential of this technique is revealed when used in a high-throughput fashion to screen up to millions of molecules, aiming to generate a sub-library rich in potential binders, thus imposing a structural filter on a given chemical library to prioritize compounds for synthesis. In high-throughput docking (HTD), where usually the protein is considered rigid or with very few degrees of freedom, two stages could be identified: (i) the prediction of the binding modes of molecules within the binding site (docking stage); (ii) the calculation of a score which attempts to predict the likelihood that a molecule will actually bind to the target. Although docking accuracy depends on the program used, the number of ligand poses with RMSD < 2 Å compared to the native structure can reach up to 80% of the studied cases (Warren et al., 2006; Wang et al., 2016). In some docking programs the binding pose is assessed by searching the global energy minimum (“docking energy”) within the potential energy surface (PES) of the protein-molecule system. Other energetic contributions should be accounted for (such as the free energy of the unbound molecule, the entropy change, and desolvation effects) in order to assign a “docking score” to molecules of a chemical library; scoring functions are classified as force-field-based, empirical, and knowledge-based (Kitchen et al., 2004). It should be highlighted that the docking energy discriminates among poses of the same molecule, while the docking score is aimed at discriminating among different molecules of the set [usually docking scores are calculated on the best pose (of few best poses) of each molecule]. In many docking programs, however, the docking score is used for both purposes.

In the last 10 years there have been continuous efforts to enhance scoring functions by incorporating some type of QM-based calculations, especially deriving system-specific charges, such as the QM-polarized ligand docking approach (Cho et al., 2005). Some degree of improvement was observed using these tailored energy functions in terms of pose prediction. However, these advances will not be addressed here, and the reader is referred to a sound review covering these issues (Mucs and Bryce, 2013).

There are fewer works describing PL interactions with explicit calculations at the QM level. One should highlight the pioneering work of Raha and Merz (Raha and Merz, 2004, 2005), who introduced QMScore, a semiempirical QM (SQM) scoring function based on the Austin Model 1 (AM1) Hamiltonian (Dewar et al., 1985), complemented with a FF dispersion term and a Poisson-Boltzmann implicit solvent model, and calculated using the linear-scaling divide and conquer method (Dixon and Merz, 1996). QMScore was able to discriminate native and decoy poses and captured essential binding affinity trends in a set of 165 PL complexes; a series of QM/MM scoring functions were also studied to discriminate native from decoy poses in six HIV-1 proteases (Fong et al., 2009), showing in some of them improvements over MM empirical potentials.

Very recently, the SQM/COSMO energy filter was introduced, aimed at discriminating native from decoy ligand docking poses (Pecina et al., 2016). The SQM/COSMO filter is a simplified version of a general binding free energy function (Raha and Merz, 2005; Lepšík et al., 2013),

(1) $$\begin{array}{r} \Delta G_{binding}=\Delta E_{\mathrm{int}}+\Delta \Delta G_{solv}+\Delta G_{conf}-T\Delta S \end{array}$$

(where Δ*E*_*int*_ is the gas-phase interaction energy, ΔΔ*G*_*sol*__v_ the solvation energy change upon complex formation, Δ*G*_*conf*_ the change of conformational free energy, and –*T*Δ*S* the entropy change upon binding). In this new filter, only the first two dominant terms in Equation (1) are conserved, thus avoiding expensive SQM optimizations. Δ*E*_*int*_ is calculated at the PM6 level (Stewart, 2007) with the D3H4X correction for dispersion, hydrogen- and halogen bonding interactions (Rezáč and Hobza, 2011, 2012), and the implicit solvent model COSMO (Klamt and Schüürmann, 1993) is used to calculate the ΔΔ*G*_*conf*_ term (this filter was named PM6-D3H4X). It was shown that calculations in a small subsystem (the ligand and neighboring amino acids) do not deteriorate results compared to the whole system, with a clear benefit in terms of computational speed. The ability of this filter to discriminate binding-like poses from decoy poses was evaluated in four challenging systems [acetyl cholinesterase (AChE), TNF-α converting enzyme (TACE), aldose reductase (AR), and HIV-1 protease (HIV PR)], and compared to seven well-known empirical scoring functions and a physics-based AMBER/GB. It was shown that the SQM/COSMO filter performed best by two metrics: the number of false-positive solutions, and the maximum ligand RMSD of all poses within a given range of a normalized score. The worst performance was on the TACE metalloprotein, containing a Zn^2+^… S^−^ interaction. As for the computational requirements, this filter is ~100 times slower than the traditional scoring functions, ~10 times slower than the AMBER/GB scoring scheme, but ~100 times faster than the standard SQM filter calculated using the full Equation (1). In a follow up contribution (Pecina et al., 2017), the SQM/COSMO filter was evaluated in the same four systems (AChE, TACE, AR, HIV PR) using the self-consistent charge density functional tight-binding (SCC-DFTB) (Elstner et al., 2001), complemented with the D3H4 corrections for dispersion and hydrogen-bond interactions (Rezáč and Hobza, 2012). This improved filter (named DFTB3-D3H4) retained its excellent performance in AChE, AR and HIV PR, and clearly improved the results on the TACE system at a reasonably higher computational price. To further validate the two variants of SQM filters, diverse 17 PL complexes were studied using the PM6-D3H4X and the DFTB3-D3H4X (extended in this case to account for halogen bonding), and compared to four standard docking programs (Ajani et al., 2017). The QM-based energy functions clearly outperformed the standard scoring functions in terms of the number of false positives.

Using MD simulations and QC energy evaluations, Burton and co-workers evaluated the preferred docking (binding) mode of the natural salpichrolide A and a synthetic analog with an aromatic D ring within the estrogen receptor α (ERα) binding site (Alvarez et al., 2015). The MM/QM-COSMO (Anisimov and Cavasotto, 2011; Anisimov et al., 2011) method with the PM6 Hamiltonian was used for the energy calculations. The MD simulations coupled with energy evaluations corresponding to different ligand-binding modes support the preferred inverted orientation of the steroids in the ERα binding site, in which the aromatic ring D occupies a similar position to the corresponding A ring of estradiol.

G protein-coupled receptors (GPCRs) present a challenging case for docking due to their solvent-exposed and polar binding sites (Cavasotto and Palomba, 2015). A new docking protocol was recently presented where a QM/MM + implicit solvation model was used to rescore docked ligand poses (Kim and Cho, 2016). The gas energy was calculated at a QM/MM level, considering the ligand and neighboring residues within 5 Å as the QM region, and the solvation energy was calculated using a Poisson-Boltzmann (PB) approach with partial charges derived from ESP fitting. Evaluating their protocol on 40 GPCR complexes including representatives of classes A, B, and F, the authors obtained an average RMSD of 0.78 Å, and a success rate of 40/40 for ligands with RMSD < 2 Å.

Chaskar et al. (2014) developed an on-the-fly QM/MM approach combining the EADock DSS docking algorithm (Grosdidier et al., 2007) with calculations based on the SCC-DFTB model and the CHARMM FF (Brooks et al., 2009), and evaluated it on a dataset of high-quality x-ray structures of zinc metalloproteins. Their method significantly improved the success rate compared to classical docking programs for orthosteric ligands in terms of ligand pose RMSD. Recently, a similar approach (Chaskar et al., 2017), but coupled with the Attracting Cavities docking algorithm (Zoete et al., 2016), was applied on three different sets: (i) the Astex Diverse data set of 85 common non-covalent drug/target complexes; (ii) a zinc metalloprotein data set of 281 complexes: (iii) a heme protein data set of 72 complexes, where ligand/protein interactions are dominated by covalent ligand/iron binding. On the first set the performance was similar to the standard scoring functions, but on the other two, QM/MM showed an improved performance, especially in the third set.

## Calculation of ligand binding free energy using quantum mechanics-based methods

The binding process of five classical AChE inhibitors was analyzed using free energy perturbation (FEP) and QM/MM MD simulations (Nascimento et al., 2017). The QM calculations were performed at the AM1 level. The Δ*G*_*binding*_ was obtained as the sum of two terms, introducing two parameters into the electrostatic and van der Waals QM/MM interaction terms in the total energy (Swiderek et al., 2014). The correlation between the experimental and calculated values was in very good agreement (*R*^2^ value of 0.96 for 100 ps simulation time). Moreover, there was a qualitative agreement of the order of inhibition between theoretical and experimental values. The use of QM to describe these ligands was of great importance due to their polar nature and the high aromaticity of the enzyme binding site.

In order to analyze the efficiency of different approaches to calculate Δ*G*_*binding*_ at the QM/MM level employing MD simulations, Ryde and Olsson have recently compared the results of the calculation of the binding of nine small carboxylate ligands to the octa-acid deep cavity host (Olsson and Ryde, 2017), via reference-potential FEP calculations (Rod and Ryde, 2005) and full QM/MM FEP simulations. The ligand was described using a SQM PM6 Hamiltonian augmented by the DH+ empirical dispersion and hydrogen-bond corrections (Korth, 2010). The results showed that the reference-potential approach is approximately three times more effective than the direct approach, and the convergence of the MM → QM/MM perturbations is improved by the addition of QM/MM MD simulations for a number of coupling parameter values between the MM and QM/MM energies.

Grimme and co-workers presented a full QM approach to evaluate absolute ligand binding free energies as the sum of three terms: the interaction energy, the solvation contribution, and the entropic term (Ehrlich et al., 2017). Calculations were performed on a reduced system consisting of the ligand and neighboring binding site atoms (~1,000 atoms in total). For the interaction energy, two methods were used: the minimal basis Hartree-Fock HF-3c (Sure and Grimme, 2013) which includes a D3 dispersion correction (Grimme et al., 2010), and the composite hybrid PBEh-3c DFT lower computational cost method (Grimme et al., 2015); entropic contributions were calculated using a semiempirical DFTB3-D3 hessian (Gaus et al., 2011; Brandenburg and Grimme, 2014); the solvation contribution was calculated with the COSMO-RS method (Klamt, 1995, 2011). Two molecular systems were studied: the activated serine protease factor X (FXa) with 25 ligands and the non-receptor tyrosine-protein kinase 2 (TYK2) with 16 ligands. The mean absolute deviation (MAD) of the Δ*G*_*binding*_ using the HF-3c level was 2.8 and 2.7 kcal/mol, with a Pearson correlation coefficient 0.47 and 0.51, respectively; while a MAD of 2.1 kcal/mol was obtained on the FXa system using the PBEh-3c method, with a Pearson coefficient of 0.53. Although the results are clearly encouraging from a QC standpoint, this approach cannot be yet used in an industrial setting, and errors stemming from the structural optimization level, conformational sampling and the solvation contribution need further development.

Frush et al. performed a QM/MM-based evaluation of Δ*G*_*binding*_ on four diverse protein targets of pharmaceutical relevance: beta-secretase 1 (BACE1), TYK2, heat shock protein 90 α (HSP90), and protein kinase R (PKR)-like endoplasmic reticulum kinase (PERK), using 22, 16, 70, and 32 ligands, respectively (Frush et al., 2017). Binding affinities were calculated using the linear interaction energy (LIE) protocol (Aqvist et al., 1994), with α and β LIE coefficients similar to those reported elsewhere (Su et al., 2007), but modified to fit the experimental affinities of the TYK2 set. Ensemble averages were calculated through QM/MM calculations on MD trajectories, describing the ligand at the SQM level using the AM1 Hamiltonian, and the rest of the system using MM. On each of the four systems, the obtained MAE was 0.86, 0.42, 0.86, and 1.11 kcal/mol, respectively, and a correlation of 0.73, 0.71, 0.60, and 0.86, respectively. The authors concluded that their methodology reached a reasonable balance between accuracy and computational cost.

In the context of the D3R grand challenge blind test competition (Gathiaka et al., 2016), Ryde and co-workers evaluated four different approaches for predicting the binding affinities of three sets of ligands of the HSP90 protein (Misini Ignjatovic et al., 2016): (i) induced-fit docking (Sherman et al., 2006) followed by calculations with three energy functions; (ii) MM/GBSA calculations on minimized docked structures; (iii) optimization of docked structures with QM/MM calculations followed by QM-based energy evaluation of a subset of ~1,000 atoms using continuous solvent; (iv) calculations of relative binding affinities using free-energy simulations. Although the results were somehow poor, the authors were able to identify the sources of error: in one case the ligand could displace water molecules (this could be found only after the experimental data was released), and for other two, ligands might exhibit alternative binding modes that those in the crystal, or conformational changes of the system might be critical.

## Conclusions and perspective

In this short review we presented the most recent applications of QM-based methods to molecular docking and ligand binding free energy prediction in the context of drug lead discovery, focusing on cases where QM is explicitly used to calculate at least some of the free energy contributions. The last 10 years have seen a remarkable interest in the development and application of QM-based methods in the field of drug discovery. This was triggered by the interest in modeling biomolecular systems in a more accurate way, and allowed by the unprecedented growth of computational power. QM methods are theoretically exact, capturing the underlying physics of the system and accounting for all contributions to the energy; thus, missing effects in FFs (such as electronic polarization, covalent-bond formation, and coupling among terms) are *de facto* accounted for in QM formulations, which are thus systematically improvable; being generally valid across the chemical space, they offer greater freedom to deal with non-standard molecules, avoiding the FF parameterizations.

Overall, the results obtained using QM approaches are very encouraging, but still different sources of error should be addressed in order to improve accuracy and predictability of these methods: (i) they are still system-dependent; thus, further validation and benchmarking are needed; (ii) in spite of the progress in computational speed, most QM applications to drug discovery cannot still be used in industrial settings, highlighting the need for optimized codes, especially those using GPUs; (iii) conformational sampling and protein flexibility: due to computing time, in most approaches aimed for high-throughput use, only local energy minimization is performed, or even no minimization at all; this should be integrated with the possibility of system cutout, and optimal combinations of these thoroughly validated; (iv) solvation contribution, especially in charges systems; (v) entropic considerations, usually omitted in many of this type of calculations. In spite of these limitations, it is clear that reliable QM methods for biomolecular systems would be a tremendous step forward toward predictive binding free energy calculations.

## Author contributions

CNC conceived, designed, and supervised this review. All authors listed have made direct and intellectual contribution to the work, and approved it for publication.

### Conflict of interest statement

The authors declare that the research was conducted in the absence of any commercial or financial relationships that could be construed as a potential conflict of interest.
